# Exploring Stakeholders' Perspectives on Required Nursing Competencies in Paediatric End‐of‐Life Care: A Qualitative Study

**DOI:** 10.1002/nop2.71011

**Published:** 2026-11-03

**Authors:** Latif Panahi, Zahra Taheri‐Ezbarami, Maryam Rassouli, Somaye Pouy

**Affiliations:** ^1^ PhD Student in Nursing, School of Nursing and Midwifery Guilan University of Medical Sciences Rasht Iran; ^2^ Department of Nursing, School of Nursing and Midwifery Guilan University of Medical Sciences Rasht Iran; ^3^ School of Nursing, College of Health Sciences University of Nizwa Nizwa Sultanate of Oman; ^4^ Cancer Research Center Shahid Beheshti University of Medical Sciences Tehran Iran; ^5^ Social Determinants of Health Research Center, Trauma Research Institute Guilan University of Medical Sciences Rasht Iran

**Keywords:** end‐of‐life care, nursing competency, paediatric nursing, palliative care, qualitative research

## Abstract

**Aim:**

To explore stakeholder perspectives (including nurses, nursing supervisors, educators and parents) on the competencies perceived as important for providing paediatric end‐of‐life care.

**Design:**

A qualitative design using semi‐structured interviews and a conventional content analysis approach was employed.

**Review Methods:**

Data were collected through individual, in‐depth, semi‐structured face‐to‐face interviews. Multi‐stakeholder purposive sampling with maximum variation was utilized. Audio‐recorded interviews were transcribed verbatim and analysed using conventional qualitative content analysis following Graneheim and Lundman's approach. Data collection and iterative analysis proceeded until code saturation was achieved within the healthcare professional cohort.

**Data Sources:**

Primary qualitative data were collected between 1 April and 30 June 2025, across paediatric healthcare settings in the North of Iran (Rasht, Guilan). Data sources comprised 17 participants: 15 healthcare professionals (10 clinical paediatric nurses, 2 nursing supervisors and 3 nursing faculty members) and 2 bereaved parents whose children had received end‐of‐life palliative care.

**Results:**

Data analysis revealed one overarching theme, ‘Providing comprehensive and ethically oriented care for the child and family at the end of life’, encompassing six stakeholder‐identified competencies (main categories) and 17 subcategories. The six proposed categories were: (1) competencies in ethical and empathetic communication; (2) competencies in comprehensive physical care focused on the child's quality of life; (3) competencies in psychological care and bereavement support; (4) competencies in cultural and spiritual care; (5) ethical and advanced clinical decision‐making competencies; and (6) competencies in interdisciplinary and team‐based collaboration.

**Conclusion:**

From the perspective of key stakeholders, competent paediatric end‐of‐life nursing care extends beyond routine clinical skills, requiring comprehensive, ethically grounded and context‐sensitive capabilities for supporting both the dying child and their family. These preliminary, stakeholder‐informed competencies highlight the need for strengthening specialized educational curricula and supportive institutional infrastructure to enhance the quality of paediatric end‐of‐life care.

**Implications for Nursing Practice:**

- *Culturally responsive care*: Highlights the need to incorporate cultural and spiritual coping strategies—such as faith in divine providence—into paediatric palliative care competencies, extending beyond Western‐oriented frameworks.
- *Navigating ethical tensions*: Equips nurses to effectively navigate complex ethical dilemmas and facilitate family‐centred decision‐making in healthcare settings characterized by limited resources and prominent religious frameworks.
- *Educational framework*: Offers an evidence‐based foundation for developing specialized undergraduate curricula, continuous professional development programs and clinical protocols for general nurses providing end‐of‐life care.

**Impact:**

This study addresses the lack of context‐specific evidence on paediatric end‐of‐life nursing competencies in Iran. The findings clarify the professional competencies required to support dignified, high‐quality end‐of‐life care, preserve children's dignity and empower families during decision‐making and bereavement. These results are anticipated to inform nurses, educators and healthcare policymakers involved in paediatric palliative care.

**Reporting Method:**

This study was reported in accordance with the Consolidated Criteria for Reporting Qualitative Research (COREQ) checklist.

**Patient or Public Contribution:**

Bereaved parents whose children passed away in paediatric units participated as key interviewees, contributing directly to qualitative data generation by sharing their lived experiences and expectations regarding nursing competencies during their child's end‐of‐life trajectory.

## 1 Introduction

End‐of‐life (EOL) care refers to a continuum of compassionate, specialized care provided to children with life‐threatening illnesses during their final days or weeks of life. In the context of this study, the EOL phase is operationally defined as the final days or weeks of a child's life when curative treatments are no longer effective or pursued, and the primary clinical objective transitions exclusively to symptom management, comfort care and holistic family support through the dying process and impending bereavement. As a specialized subset of paediatric palliative care, paediatric EOL care aims to manage physical distress, enhance the quality of the child's death and support the family throughout the grief trajectory (Haavisto et al. 2021). EOL care adopts a patient‐ and family‐centred approach that simultaneously addresses the physical, psychological, social and spiritual dimensions of care (Nacak and Erden 2022).

According to global estimates, approximately 21 million children worldwide require palliative care services, with nearly 7 million dying each year (Connor et al. 2017; United Nations Inter‐agency Group for Child Mortality Estimation 2020). Nevertheless, access to paediatric EOL care remains highly inequitable across regions, and in many settings, the absence of structured care delivery systems poses formidable challenges (Clelland et al. 2020; Fraser et al. 2020). Based on the EMRO Atlas (2020), Iran's paediatric palliative care capacity is currently categorized at the ‘capacity building’ stage (World Health Organization. Regional Office for the Eastern Mediterranean 2023).

In many instances, paediatric deaths occur in acute hospital settings under intensive medical management, often leaving families with heightened uncertainty regarding the quality of EOL care provided (Eltaybani et al. 2021). Such high‐stress environments can reduce family involvement in decision‐making, increase moral distress among healthcare providers and ultimately compromise care quality (Hao et al. 2021). Consequently, EOL care involves complex ethical and emotional challenges, making it a critical focus for healthcare policymakers and nurse executives (Hao et al. 2021).

Despite socio‐cultural and structural variations across healthcare systems, supporting parents during this critical phase is recognized as a cornerstone of paediatric EOL care worldwide (Koch and Jones 2018). Beyond profound emotional trauma, parents face demanding caregiving responsibilities and ethically distressing decisions, such as the withdrawal or withholding of life‐sustaining treatment (Chambers 2018). Therefore, care delivery must extend beyond the child to empower parents to maintain their caregiving role while receiving tailored bereavement support (Barrett et al. 2023). Indeed, evidence highlights that affected children and their informal caregivers encounter extensive unmet holistic needs—particularly encompassing physical, psychological and end‐of‐life care dimensions—which are often compounded by infrastructural deficits and limited family empowerment (Pouy et al. 2023). Quality EOL care yields substantial benefits, including the timely identification of patient needs, alleviation of distress and optimized health resource utilization, proving both clinically and economically effective (Ghaemizade Shushtari et al. 2022; Mota‐Romero et al. 2021; Worldwide Palliative Care Alliance and World Health Organization 2014).

The concept of a ‘good death’ in paediatric palliative care denotes a death characterized by the preservation of human dignity, effective symptom control and the fulfilment of the child's emotional and spiritual needs. Given children's unique developmental vulnerabilities and the sensitive nature of family communication, achieving a ‘good death’ is directly linked to high‐quality nursing care (Broden et al. 2020). The overarching goal of EOL care is to facilitate a good death by managing distress and establishing a peaceful, supportive environment for both the child and their family (Chong et al. 2019).

Nurses play a pivotal role in delivering EOL care (Taheri‐Ezbarami et al. 2024), a responsibility widely acknowledged as one of the most emotionally demanding aspects of clinical practice (Clark et al. 2014; Wong et al. 2022). Although skilled nursing can foster positive, meaningful memories for grieving families (Harris et al. 2014), many paediatric patients in hospital settings worldwide still do not receive adequate evidence‐based EOL care (Connolly et al. 2016). A key contributing factor is the lack of specific nursing competencies in paediatric EOL care (Strautmann et al. 2020).

Professional competence comprises an integrated combination of knowledge, clinical skills, attitudes, values and judgment essential for delivering safe, high‐quality care (Gamondi et al. 2013; Griffith 2018; Malik and Chapman 2017; Thavaraj and Gillett 2019).

However, evidence demonstrates that many nurses, particularly in developing countries, feel ill‐prepared to deliver appropriate EOL care (Anstey et al. 2016; Ghaemizade Shushtari et al. 2022). Caring for dying children requires advanced specialized competencies that are rarely covered in undergraduate or postgraduate nursing curricula (Aziz et al. 2012; Taheri‐Ezbarami et al. 2024). Furthermore, most existing competency frameworks are tailored for specialized palliative care practitioners, neglecting general paediatric nurses who routinely provide frontline EOL care (Clark et al. 2014; Gamondi et al. 2013; Jeffers et al. 2022). In Iran, paediatric EOL care is not yet formally institutionalized as a distinct specialty, leaving many general nurses without formal training or guidance in this critical area of practice.

Critically, existing international competency models present specific conceptual and contextual limitations when applied to paediatric settings in developing countries (Connolly et al. 2016; Gamondi et al. 2013). First, established international educational frameworks often presume the presence of dedicated palliative care teams and robust institutional infrastructures (Postier et al. 2022), overlooking the operational reality of resource‐constrained settings where general paediatric nurses must independently shoulder end‐of‐life responsibilities without structured protocols (Ghaemizade Shushtari et al. 2022). Second, existing frameworks predominantly prioritize individual patient autonomy and formal advance directives, offering inadequate practical guidance for navigating collectivistic, family‐centred decision‐making and religious coping mechanisms that strongly characterize paediatric dying trajectories in Middle Eastern and Islamic contexts (Eltaybani et al. 2021; Ghaemizade Shushtari et al. 2022). Consequently, general nurses frequently experience profound moral distress when standard clinical guidance conflicts with entrenched cultural realities. To overcome these limitations, the empirical contribution supported by this study's multi‐stakeholder data is threefold: First, it captures the frontline, operational competencies required by general paediatric nurses operating without specialized palliative team support. Second, by triangulating perspectives of bedside nurses, nurse leaders, educators and bereaved parents, this study reveals how ethical communication, emotional labour and clinical judgement must be adapted to honour religious‐cultural nuances (e.g., integrating spiritual solace into clinical care and facilitating parental coping with treatment limitation). Third, it empirically delineates the boundary between individual professional competencies and overarching organizational deficits, thereby identifying actionable, context‐sensitive competencies that can directly inform undergraduate curricula and in‐service training programs in nascent palliative care systems.

To address this knowledge gap, this study aimed to explore stakeholder‐perceived competencies for paediatric EOL care within a context‐sensitive framework. In this study, nursing competency is operationally conceptualized through stakeholders' accounts as the dynamic integration of knowledge, clinical skills, professional attitudes and ethical reasoning perceived as necessary to deliver high‐quality, family‐centred care. To ensure conceptual clarity, a clear distinction is made between individual nursing competencies and the underlying structural or organizational conditions. Conceptually, nursing competency is shaped by the dynamic interplay between personal capabilities and supportive practice environments. Conversely, organizational constraints (e.g., inadequate staffing, lack of formal protocols, or environmental deficits) represent contextual barriers rather than individual competencies. Therefore, the categories analysed in this study focus specifically on stakeholders' perceptions of nurses' clinical capabilities, decision‐making and professional judgements, distinct from routine nursing duties or organizational deficiencies. Ultimately, this qualitative study investigated the core competencies perceived as essential for paediatric EOL care from the perspectives of bedside nurses, nurse leaders, educators and parents.

## 2 The Study

### 2.1 Aim

The present study aimed to explore stakeholder perspectives—including bedside nurses, nurse leaders, academic faculty and parents—regarding the core competencies perceived as necessary for delivering paediatric EOL care.

## 3 Methods

### 3.1 Design

This study employed a qualitative design with a conventional content analysis approach to explore stakeholder perspectives on the competencies perceived as important for nurses providing EOL care for paediatric patients. Given the scarcity of context‐specific and culturally grounded evidence on paediatric EOL nursing competencies in Iran, a qualitative approach was deemed appropriate to obtain an in‐depth understanding of participants' lived experiences and perspectives. The study is reported in strict accordance with the Consolidated Criteria for Reporting Qualitative Research (COREQ) guidelines (Tong et al. 2007).

### 3.2 Participants

The study included 17 participants, comprising paediatric clinical nurses, nursing managers/supervisors, nursing faculty members and parents of paediatric patients receiving EOL care. A multi‐stakeholder purposive sampling strategy with maximum variation was employed to capture a 360‐degree perspective of the phenomenon. Integrating these diverse groups allowed the exploration of paediatric end‐of‐life competencies across clinical, administrative, educational and familial vantage points, thereby developing a preliminary, context‐informed conceptualization. Although the overarching themes reflect shared perspectives, group‐specific nuances are explicitly highlighted in the findings. Specifically, the primary focus remained on professional and clinical competencies identified by healthcare professionals; however, parental feedback was deliberately incorporated to frame these competencies from a family‐centred viewpoint. The inclusion of parents was not intended to evaluate parental competencies but rather to serve as source triangulation, capturing the lived experiences of care recipients and identifying the practical, emotional and relational capabilities they perceive as important and expect from nurses.

To differentiate paediatric EOL care from broader paediatric palliative care (PPC) (Linebarger et al. 2022; World Health Organization 2018), the EOL phase in this study was operationally defined as the period when life‐sustaining and curative treatments are no longer deemed effective. During this phase, the primary clinical focus shifts entirely to comfort, symptom management and preparation for death, typically encompassing the final days to weeks of life, active dying and immediate bereavement (Linebarger et al. 2022; Villanueva et al. 2016). The determination of the EOL phase was made by attending paediatricians and the multidisciplinary Paediatric Intensive Care Unit (PICU) team based on clinical prognosis and treatment limitation decisions. Consequently, eligibility for clinical participants required specific, direct experience in managing patients during this active dying phase, rather than merely providing care for stable life‐limiting conditions.

### 3.3 Inclusion and/or Exclusion Criteria

For clinical nurses and supervisors, inclusion criteria comprised willingness to participate and a minimum of 6 months of direct clinical experience caring for paediatric patients at the end of life. For nursing faculty members, eligibility required formal experience in teaching theoretical or clinical content related to paediatric EOL care. For parents, inclusion criteria were: being a bereaved parent whose child had a confirmed life‐limiting or life‐threatening illness and had received active inpatient paediatric end‐of‐life care prior to death; the interview took place within 4 to 8 months' post‐loss (a timeframe ethically established to avoid acute distress during immediate grief while ensuring vivid, reflective recall of bedside nursing competencies); and willingness to share their experiences.

Exclusion criteria across all participant groups included a lack of willingness to participate and current, acute severe psychological distress or active psychiatric conditions (e.g., severe acute bereavement crisis or active clinical depression) that could impair informed consent capacity or heighten vulnerability to emotional harm during in‐depth interviews. This was evaluated via self‐report and clinical judgement during initial screening to uphold the ethical principle of non‐maleficence.

### 3.4 Study Setting and Recruitment

The study was conducted in a tertiary paediatric referral teaching hospital and an affiliated nursing faculty in a metropolitan region. The hospital serves as a regional referral centre for children with life‐limiting and life‐threatening conditions. Paediatric EOL care is predominantly provided in the PICU due to symptom complexity and the need for specialized care. Additionally, when paediatric oncology or general ward patients experience clinical deterioration, nurses in those units are actively involved in EOL care. These settings were selected due to their high concentration of relevant clinical cases, experienced paediatric nursing staff and suitability for generating rich qualitative data.

To ensure psychological safety during these sensitive interviews, robust protocols were established. Participants were interviewed in private, quiet and comfortable settings, and were explicitly informed of their right to pause or terminate the interview at any point without penalty. Furthermore, all participants were provided with direct access to institutional psychological support services if emotional distress occurred.

### 3.5 Researcher Characteristics and Reflexivity

All interviews were conducted by the first author, a female Assistant Professor of Nursing holding a PhD and specialized fellowship training in palliative care, with extensive qualitative research experience. Importantly, the first author held no clinical, managerial, educational, or therapeutic authority over any participants, minimizing power imbalances and social desirability bias. While some academic colleagues were acquainted with the researcher, strict assurances of confidentiality and anonymity were established prior to each interview.

Given the principal investigator's specialized background in palliative care, there was an inherent risk of pre‐existing assumptions influencing data interpretation. To maintain reflexivity and mitigate potential bias, the researcher actively practiced bracketing throughout the study. This was operationalized through detailed reflective field notes written immediately after each interview and analytical memos maintained during coding, ensuring that final categories were authentically grounded in raw participant accounts rather than preconceived professional ideals.

### 3.6 Data Collection

Potential participants were approached face‐to‐face by the principal investigator in clinical and academic settings, where study objectives were presented prior to formal invitation. Of 20 individuals approached, three declined due to clinical workload and time constraints, resulting in a final sample of 17 participants. A multi‐stakeholder purposive sampling strategy with maximum variation was employed. Maximum variation was explicitly sought across clinical departments (PICU, Internal ward and Oncology), years of clinical experience and professional roles to capture a diverse, multi‐stakeholder perspective.

Data were collected between 1 April and 30 June 2025, using individual, semi‐structured, in‐depth interviews conducted in quiet, private rooms chosen based on participant preference. Written informed consent was obtained prior to data collection and audio recording. Participation was entirely voluntary. Interview duration ranged from 35 to 75 min (mean: 52 min; median: 48 min).

An initial interview guide was developed by the research team‐based on literature and study objectives. To evaluate clarity and refine the questioning strategy, two pilot interviews were conducted with eligible paediatric clinical nurses. These pilot participants met all inclusion criteria but were excluded from the final study sample. The pilot sessions revealed that initial theoretical questions were difficult for bedside clinicians to answer directly. Consequently, the interview guide was restructured to elicit concrete, narrative clinical accounts (e.g., describing a specific EOL care scenario rather than abstractly defining ‘competency’). Because these structural adjustments altered interview dynamics, pilot data were excluded from the final analysis and saturation assessment. The finalized interview guide is provided in Appendix S2.

To optimize data richness, the first participant was purposefully selected based on extensive clinical experience in paediatric EOL care to provide a foundational narrative. Concurrent data collection and preliminary analysis guided subsequent sampling to explore emerging variations. This subsequent selection was driven by ‘analytical needs’ to ensure depth and variation within the emerging categories—a standard practice in conventional qualitative content analysis, rather than formal theoretical sampling grounded in Grounded Theory.

Data collection continued until code saturation was systematically achieved within the primary healthcare professional cohort (*n* = 15; comprising 10 clinical bedside nurses, three nursing faculty members and two nursing supervisors). Code saturation was operationalized as the point at which no new codes, novel conceptual properties, or distinct subcategories emerged from subsequent transcripts (Hennink and Kaiser 2022). By the 13th interview, conceptual recurrence was evident across all core competency domains, and the subsequent two interviews (14 and 15) yielded no novel descriptive codes or properties, confirming code saturation and structural stability of the coding framework. The remaining two interviews with bereaved parents (interviews 16 and 17) were conducted not to achieve independent subgroup saturation, but rather to ensure code stability, provide critical multi‐stakeholder triangulation, and contextually ground the stakeholder‐identified competencies within the lived experiences of families (specifically validating the experiential significance of compassionate presence, dignified post‐mortem handling, and non‐abandonment without introducing structurally disparate codes).

Sample interview questions included:

- For Nurse Leaders: ‘Drawing from your expertise, what are the primary clinical responsibilities of a skilled nurse in pediatric EOL care?’/‘Which nursing interventions provide the greatest benefit to children and families during the terminal phase?’
- For Bedside Nurses: ‘Could you describe your clinical experiences in delivering pediatric EOL care?’/‘What core competencies are essential for nurses providing care during this phase?’
- For Faculty Members: ‘Based on your academic observation, what are the key competencies required for nurses delivering pediatric EOL care?’/‘How do you evaluate current nursing practice about pediatric EOL care?’
- For Parents: ‘Could you share your experience regarding the nursing care your child received?’/‘Which aspects of nursing support were most helpful to you and your family during this time?’

Probing questions (e.g., ‘Could you provide a specific clinical example?’ or ‘Could you elaborate further on that aspect?’) were utilized to deepen participant narratives.

### 3.7 Data Analysis

For data management and analysis, we utilized MAXQDA 2022 software and applied conventional qualitative content analysis following Graneheim and Lundman's (2004) steps (Graneheim and Lundman 2004). The first author conducted the initial open coding for all 17 transcripts. To ensure analytical rigour, three early transcripts (approximately 18% of the dataset) were independently coded by a second researcher experienced in qualitative methods. Based on these initial transcripts, a dynamic coding framework was developed in MAXQDA, which was continuously revised as new data emerged.

Each interview transcript served as an analytical unit. The primary researcher identified meaning units and condensed them into approximately 580 initial codes. Subsequently, the entire dataset—including the complete coding structure and all extracted meaning units—was systematically reviewed by the wider research team. Coding disagreements were identified during bi‐weekly analysis meetings. Rather than relying on superficial consensus, disagreements were resolved by returning to the raw transcripts and audio recordings to re‐contextualize the quotes. If differing interpretations persisted, codes were refined or split. All decisions to merge, separate, or rename categories were rigorously documented using MAXQDA's memo‐writing and logbook features to maintain a transparent analytical audit trail.

Through constant comparison, the 580 initial codes were condensed into 17 subcategories and six categories. For example, a subcategory initially named ‘Responsive Acknowledgment’ was later enhanced and renamed ‘Emotional Empathy and Active Listening’ to better reflect its constituent codes. The overarching main theme of the study was identified collaboratively by the full research team during a dedicated final analysis workshop, where researchers abstracted the descriptive categories into a comprehensive core meaning. Finally, two external qualitative researchers conducted a peer‐debriefing review. These external reviewers were provided with the final coding framework, the analytical audit trail and three full, anonymized transcripts to rigorously cross‐check the logical progression from raw data to the overarching theme.

We conducted a cross‐stakeholder exploratory analysis as a crucial part of our analysis to gain a full understanding of the phenomenon. The four stakeholder groups were analysed together because paediatric EOL care is inherently multiperspective. While bedside nurses provided the clinical experiences, nursing supervisors offered insights into managerial expectations, faculty members highlighted educational ideals, and parents grounded these perceived competencies in the actual needs and lived experiences of families. During the inductive analysis, we systematically examined how shared competencies were described across roles, while noting descriptive variations in emphasis. Because subgroup sample sizes were uneven, these cross‐stakeholder observations are presented descriptively for triangulation and contextual nuance rather than as formal subgroup comparative evidence. Distinct parental contributions were carefully evaluated to triangulate and enhance the understanding of themes predominantly identified by professionals. In the Findings section, we explicitly report these cross‐group dynamics for each category, specifying whether a competency was supported across stakeholder groups or was primarily derived from one group. The analysis kept its focus on the study's primary objectives, ensuring all voices were included in the data.

Categories and subcategories were directly derived from and named based on the interview‐generated codes, in line with the nature of conventional content analysis. According to Graneheim and Lundman's methodology, the phases of content analysis and their associated actions are illustrated in Table 1.

**TABLE 1:** Steps of conventional content analysis based on Graneheim and Lundman (2004).

Proposed step	Actions taken in the present study
Gaining a sense of the whole	The interview transcripts were read and reread several times by the researchers to obtain a comprehensive understanding of the data.
Identifying the unit of analysis	All interview transcripts were considered as the unit of analysis.
Identifying meaning units	Words, sentences and paragraphs related to the study aim were identified as meaning units.
Condensing meaning units	Meaning units were condensed while preserving their core meaning.
Coding	Condensed meaning units were labelled with initial codes.
Categorization	Initial codes were compared based on similarities and differences and grouped into categories and subcategories.
Abstraction and theme development	Through an inductive process, categories and subcategories were abstracted, and underlying meanings were interpreted to generate the main themes.

In addition, an instance of the process of extracting codes, subcategories and categories from participants' quotations is illustrated in Table 2.

**TABLE 2:** Example of data analysis.

Raw data/quotation	Meaning unit	Initial code	Subcategory	Category	Overarching theme
‘We received sincere responses from the nurse about the prognosis after she sat with us. She presented the facts with clarity and kindness, avoiding false hope, which empowered us to make the best decision for our child.’	Communicating honestly and clearly with parents about their child's prognosis, without misleading them, to aid their decision‐making.	Honest information disclosure	Applying communication skills for ethical truth‐telling and information sharing	Competencies in ethical and empathetic communication	Providing comprehensive and ethically oriented care for the child and family at the end of life
‘When the family and medical staff disagreed on future actions, the nurse facilitated a discussion to address worries, promote mutual understanding, and reach a joint decision benefiting the child.’	The effective navigation of disputes between families and medical staff concerning a child's serious healthcare needs.	Managing expectations	Applying conflict‐resolution skills in managing family expectations	Competencies in ethical and empathetic communication	

To preserve contextual and cultural authenticity, primary data collection and analysis (transcription, open coding, categorization) were conducted in Persian.

To ensure analytical rigour and prevent coding overlap between conceptually adjacent subcategories, specific operational decision rules were established during the iterative coding process:

(a) Managing Family Expectations (Subcategory 1.2) versus Ethical Dilemmas and Conflicts (Category 5): Text units were coded under Subcategory 1.2 when the conflict arose from parental grief reactions, emotional non‐acceptance, or communication misalignments regarding the realistic prognosis, and where resolution relied on interpersonal de‐escalation and expectations alignment. Conversely, units were assigned to Category 5 when the tension centred on fundamental moral dilemmas, surrogate decision‐making conflicts (e.g., parental demands for non‐beneficial aggressive resuscitation versus clinicians' judgment of medical futility), or legal/institutional mandates governing treatment limitation.

(b) Emotional Empathy (Subcategory 1.3) versus Bereavement Support (Category 3/Subcategory 3.2): Units were coded under Subcategory 1.3 when active listening, compassionate presence and emotional attunement served as ongoing relational tools while the child was alive and undergoing active deterioration. In contrast, units were assigned to Category 3 when supportive interpersonal behaviours were temporally situated during the peri‐mortem or post‐mortem trajectory, specifically aimed at navigating parental grief, facilitating farewell rituals, or providing structured post‐loss solace.

These operational demarcations were logged in the codebook and systematically cross‐checked during bi‐weekly peer consensus meetings to maintain coding consistency.

### 3.8 Translation Procedures

A rigorous forward‐backward translation method was utilized for illustrative direct quotes. First, a bilingual researcher translated selected Persian quotes into academic English. Second, an independent bilingual expert back‐translated the English text into Persian to verify conceptual equivalence. Discrepancies were resolved through team consensus. Special care was taken to preserve culturally and religiously sensitive concepts (e.g., spiritual hope, dignity, death rituals). Minor grammatical edits were applied to English translations solely to enhance readability (e.g., removing conversational fillers) while preserving original meaning and emotional tone.

### 3.9 Rigour and Trustworthiness

Study rigour and trustworthiness were established using Lincoln and Guba's evaluative criteria (Credibility, Dependability, Confirmability, Transferability and Authenticity) (Mota‐Romero et al. 2021). Data credibility was improved through regular discussions with the research team during data analysis. These meetings involved joint review and discussion of meaning units, initial codes, subcategories and categories. We discussed and agreed on how to handle any unclear or conflicting data interpretations. Additionally, the researchers' in‐depth involvement with the data, characterized by sufficient time dedicated to its collection and analysis, and iterative examination of interview transcripts, facilitated a more profound grasp of the participants' lived experiences.

Data dependability was achieved through purposive sampling with maximum variation across work experience, workplace setting and paediatric EOL care experience. Moreover, the research team and external reviewers scrutinized the data analysis and analytical choices throughout the project to enhance the consistency and stability of the analytical framework.

Data confirmability was strengthened through systematic documentation of all study phases, from data collection to theme extraction, and a transparent record of the analytical decision trail. Furthermore, to establish the accuracy of the data, transcript verification was utilized; the verbatim transcripts were returned to 6 participants (2 nurses, 2 nursing faculty members and 2 parents) to confirm transcription fidelity. No substantive changes were requested. To validate our interpretations and further enhance credibility (true member checking), a summary of the preliminary codes and emerging categories was shared with 4 participants. Their feedback confirmed that the preliminary findings authentically represented their experiences and assisted the research team in refining the terminology of certain subcategories, ensuring that transcript checking was not conflated with thematic validation.

To ensure data transferability, the research context, participant details, and verbatim quotes in the findings were thoroughly described. This helps readers judge if the results fit other situations.

Finally, to maintain data authenticity, the researchers aimed to accurately reflect participants' experiences and perspectives. By preserving the original tone and meaning of the participants' accounts, the researchers ensured that readers could profoundly connect with and experience the described situations themselves.

### 3.10 Ethical Considerations

The study protocol was approved by the Institutional Ethics Committee of Guilan University of Medical Sciences (IRB approval: IR.GUMS.REC.1403.497, dated 22 January 2025) and conducted in accordance with the Declaration of Helsinki. Informed written consent was obtained from all participants prior to data collection.

Given the vulnerability of families in EOL care, dedicated ethical safeguards were implemented. Clinical treating teams were strictly excluded from parent recruitment to prevent perceived coercion. Parent recruitment was handled independently, with explicit assurances that participation decisions would not impact their child's medical care. Parental interviews were conducted during stable palliative care phases, avoiding moments of acute clinical crisis. Interviewers followed a formal distress protocol; if emotional distress occurred, interviews were paused and participants were offered immediate support or referral to dedicated hospital clinical psychologists and social workers. Pseudonyms were assigned to ensure complete anonymity, and all digital data were stored securely in encrypted files.

## 4 Findings

A total of 17 participants took part in the study, including 10 clinical nurses, two nursing supervisors, three nursing faculty members, and two parents of children in the end stages of life. The mean age of the participants was 38.82 years (SD = 6.10). Among the 15 healthcare professional participants, the mean work experience was 10.33 years (SD = 4.10; range: 5–20 years). The demographic characteristics of the participants are presented in Table 3.

**TABLE 3:** Demographic characteristics of participants (*N* = 17).

No.	Education	Gender	Age (year)	Marital status	Work experience (year)	Position
1	MSc	Female	36	Married	8	PICU Nurse
2	MSc	Female	38	Married	12	PICU Nurse
3	PhD	Female	39	Married	8	Assistant Professor
4	PhD	Female	42	Single	7	Assistant Professor
5	BSc	Female	37	Married	12	Oncology Nurse
6	MSc	Female	52	Married	16	Nursing Supervisor
7	MSc	Female	40	Single	13	Oncology Nurse
8	BSc	Female	36	Married	9	Nursing Supervisor
9	BSc	Female	33	Married	8	Oncology Nurse
10	BSc	Female	36	Single	12	Nurse (Internal ward)
11	BSc	Female	28	Married	6	PICU Nurse
12	BSc	Female	44	Married	14	PICU Nurse
13	BSc	Female	30	Married	4	Instructor
14	BSc	Female	48	Single	19	Nurse (Internal ward)
15	BSc	Female	37	Married	7	Nurse (Internal ward)
16	BSc	Female	39	Married	—	Bereaved Mother
17	Diploma	Female	45	Married	—	Bereaved Mother
Mean ± SD		—	38.82 ± 6.10	—	10.33 ± 4.10 (range: 5–20)	—

Abbreviation: PICU, pediatric intensive care unit.

Following conventional qualitative content analysis, a total of 580 initial codes were extracted and systematically abstracted into six primary stakeholder‐identified competencies (main categories) and 17 constitutive behavioural expressions and clinical practices (sub‐categories). All main categories converged into one overarching theme: ‘Providing comprehensive and ethically oriented care for the child and family at the end of life’. This theme reflects the participants' holistic perspectives on paediatric EOL care, triangulating insights from clinical nurses, nursing supervisors, academic faculty and bereaved parents. In this conceptualization, the six overarching categories reflect broad competency areas—integrating specialized knowledge, clinical judgement and professional values—while the subcategories delineate the concrete behaviours, communicative actions and practice indicators through which nurses enact these competencies at the bedside. Rather than presenting a validated competency framework, these findings provide a preliminary, context‐sensitive conceptualization of perceived paediatric EOL nursing competencies. The comprehensive hierarchy of competencies and behavioural expressions is summarized in Table 4.

**TABLE 4:** The overarching theme, categories and sub‐categories extracted from the data.

Main category	Category	Subcategory
Providing comprehensive and ethically oriented care for the child and family at the end of life	Competencies in ethical and empathetic communication	Applying communication skills for ethical truth‐telling and information sharing
		Applying conflict‐resolution skills in managing family expectations
		Demonstrating emotional empathy and active listening
	Competencies in comprehensive physical care focused on the child's quality of life	Demonstrating clinical proficiency in comprehensive and continuous symptom assessment
		Demonstrating proficiency in non‐pharmacological comfort interventions
		Demonstrating educational skills for family empowerment
	Competencies in psychological care and bereavement support	Applying psychological support skills to enhance family resilience
		Demonstrating supportive interpersonal skills during family bereavement
		Assessing needs and navigating professional boundaries
	Competencies in cultural and spiritual care	Demonstrating spiritual care skills to facilitate meaning‐making and comfort
		Tailoring care plans to families' religio‐cultural beliefs, modesty traditions and taboos
		Applying clinical judgement for targeted spiritual and cultural referrals
	Ethical and advanced clinical decision‐making competencies	Demonstrating facilitation skills in shared decision‐making
		Applying ethical judgement and conflict‐resolution skills in EOL dilemmas
		Applying dignity‐conserving care practices
	Competencies in interdisciplinary and team‐based collaboration	Demonstrating collaborative teamwork and communication
		Demonstrating effective information exchange skills for continuity of care

### 4.1 Providing Comprehensive and Ethically Oriented Care for the Child and Family at the End of Life

This study defines competency as the dynamic integration of knowledge, skills, attitudes, ethical judgement, values and observable professional behaviours perceived by stakeholders as essential for providing safe, dignified and effective EOL care to children and their families. The findings revealed that perceived nursing competencies in paediatric EOL care extend substantially beyond technical‐clinical interventions, encompassing communication, clinical‐ethical reasoning, cultural and spiritual attunement, holistic physical care, bereavement support and interprofessional teamwork.

#### 4.1.1 Conceptual Boundaries and Interrelatedness of Categories

Although EOL paediatric nursing care is inherently integrative and competencies often intersect in clinical practice, each identified category represents a distinct conceptual and behavioural area to avoid redundant evaluation. Specific conceptual boundaries were delineated across intersecting areas:

- *Communication (Category 1)* versus *ethical decision‐making (Category 5)*: Category 1 encompasses the interpersonal process, communicative techniques and emotional mediation required to navigate difficult interactions (the ‘how’ of communication). In contrast, Category 5 addresses the ethical frameworks, legal mandates and clinical judgment required to establish care goals, manage moral distress and enact treatment limitations (the ‘what’ of clinical resolution).
- *Cultural and spiritual care (Category 4)* versus *communication and team collaboration (Categories 1 & 6)*: Category 4 is bounded by the substantive content of existential, religious and worldview‐specific needs (e.g., accommodating faith rituals, existential meaning‐making), whereas Categories 1 and 6 delineate the relational vehicles and systemic care‐coordination mechanisms through which these individualized needs are expressed and operationalized across the interprofessional team.
- *Preserving dignity and family expectations*: While dignity and expectation management are foundational themes across palliative care, within this framework, expectation management is situated as a relational/communicative competence (Category 1), and dignity preservation is operationalized as an ethical‐clinical standard in end‐of‐life decision‐making and bedside interventions (Category 5).

## 5 Category 1: Competencies in Ethical and Empathetic Communication

One of the categories of nursing competencies in paediatric EOL care was the ability to establish honest, empathetic and culturally attuned communication with families. This category requires specific knowledge, skills and ethical judgments, categorized into three subcategories.

### 5.1 Multi‐Stakeholder Perspectives and Contextual Nuances

All participating groups viewed ethical communication and empathy as foundational elements of palliative care; however, their emphasis reflected their specific functional vantage points. Bedside nurses highlighted the emotional toll and practical pacing required when delivering bad news. Nursing supervisors viewed communication primarily as an operational mechanism for conflict mitigation and reconciling mismatched expectations. Academic faculty framed this competency around formal bioethical principles (e.g., veracity, non‐maleficence) and structured communicative models. Meanwhile, within the narrative accounts of the participating parents, emphasis was placed on receiving compassionate, transparent and jargon‐free updates, alongside nurses' genuine presence and capacity for unhurried, active listening during critical clinical turning points.

#### 5.1.1 Applying Communication Skills for Ethical Truth‐Telling and Information Sharing

This competency involves the knowledge of the family's health literacy and emotional readiness, and the communication skills required for truth‐telling in paediatric EOL care. Participants emphasized that truth‐telling should be delivered with careful consideration of the family's level of health literacy, age and psychological readiness. As a professional attitude, nurses are expected to communicate realities without concealment or avoidance, while using compassionate, clear and context‐appropriate language. The clinical judgment in this competency plays a pivotal role in building trust and preventing emotional collapse. A PICU nurse detailed this sensitive balance:

…The nurse has to tell the truth, but in a way that does not cause the family to completely collapse psychologically. You cannot just drop harsh clinical realities abruptly; you must assess their readiness, sit down with them, choose compassionate words, and know exactly how, when, and how much to share in each conversation… (P1, PICU Nurse).

A bereaved mother reinforced the necessity of gradual, compassionate honesty:

…We knew our child was slipping away, but having the nurse explain test results gently, without cold medical terms, helped us face reality without losing all our strength… (P16, Bereaved Mother).

#### 5.1.2 Applying Conflict‐Resolution Skills in Managing Family Expectations

This competency requires advanced conflict‐resolution skills and ethical judgment. Nurses play a crucial mediating role in moderating unrealistic family expectations and facilitating mutual understanding between the family and the healthcare team. This competency is particularly important in sensitive EOL situations, such as navigating the transition from curative to palliative care. A nursing supervisor highlighted this mediating role:

…Families often demand continuous aggressive resuscitation because they feel that stopping means giving up on their child. In these agonizing moments, experienced nurses calmly explain the physiological realities and the purpose of comfort care, helping parents transition from unrealistic treatment hopes toward protecting their child from unnecessary suffering… (P8, Nursing Supervisor).

#### 5.1.3 Demonstrating Emotional Empathy and Active Listening

This subcategory highlights nurses' communication skills, specifically the ability to deeply understand families' emotional experiences, engage in active listening without interruption, provide a genuine presence, and attend simultaneously to the emotional needs of both mothers and fathers. It reflects an empathetic professional attitude. A mother of a child with advanced malignancy shared:

…When the doctors told us no further curative treatments were possible, the world stopped. A nurse took my husband and me into a private room, held my hand, and said, ‘Cry as much as you need; I am right here with you.’ She stayed and listened to our fears for nearly an hour without rushing us… (P16, Bereaved Mother).

An oncology nurse corroborated the clinical significance of this presence:

…Active listening is not passive silence; it is holding space for parental anger and despair without being defensive, letting them express their deepest pain… (P7, Paediatric Oncology Nurse).

## 6 Category 2: Competencies in Comprehensive Physical Care Focused on the Child's Quality of Life

Physical care for children at the end of life extended beyond symptom control and was primarily oriented toward enhancing the child's remaining quality of life. This category encompasses the clinical knowledge and skills necessary for comprehensive physical care.

### 6.1 Multi‐Stakeholder Perspectives and Contextual Nuances

There was broad consensus among participants that meticulous symptom management and improving the child's quality of life are non‐negotiable priorities. Yet, the practical translation of this competency varied. Clinical nurses focused on continuous, 24/7 symptom monitoring and providing hands‐on bedside coaching to parents for medication and positioning. Faculty members championed the integration of evidence‐based non‐pharmacological modalities (such as comfort measures) and structured adult‐learning principles. Nursing supervisors highlighted the necessity of safe, interprofessional palliative care protocols. For parents, the true value of this competency was empowerment; they deeply appreciated nurses who taught them practical skills to actively soothe and comfort their child.

#### 6.1.1 Demonstrating Clinical Proficiency in Comprehensive and Continuous Symptom Assessment

Nurses were required to possess advanced clinical skills in assessing pain, respiratory distress, weakness and other common symptoms to prevent unnecessary suffering. According to participants, having the knowledge and skill for comprehensive symptom assessment formed the foundation for appropriate care delivery and effective alleviation of the child's discomfort. A nursing supervisor emphasized:

…Pediatric symptom assessment at the end of life cannot be routine or intermittent; it demands continuous clinical intuition. I assess the child's breathing patterns, skin breakdown, and facial tension constantly to ensure our pain and symptom protocols are adjusted before the child experiences overt suffering… (P8, Nursing Supervisor).

#### 6.1.2 Demonstrating Proficiency in Non‐Pharmacological Comfort Interventions

This subcategory highlights the specific clinical skills and knowledge required to manage distressing symptoms beyond pharmacological approaches. Competent nurses do not merely perform routine comfort measures; rather, they use clinical reasoning to select, tailor and safely apply non‐pharmacological interventions (e.g., positioning, touch, or relaxation techniques) based on the unique trajectory of the dying child. A nursing faculty member explained:

…Medications alone are insufficient for holistic comfort. During clinical training, we demonstrate how simple, non‐invasive measures—such as elevating the head of the bed, using specialized gel cushions, or applying cool cloths—significantly relieve respiratory distress and agitation in terminal stages. Nurses must master these bedside comfort arts… (P4, Nursing Faculty).

#### 6.1.3 Demonstrating Educational Skills for Family Empowerment

This subcategory moves beyond routine information delivery to emphasize the communication skills and educational competencies needed at the end of life. Competency in this area involves the ability to assess the family's health literacy, emotional readiness and information needs. By utilizing tailored educational skills, competent nurses empower families to participate meaningfully in decision‐making and caregiving during their child's final moments. A clinical nurse noted:

…When a child is approaching the terminal stage, we actively train parents at the bedside. I show mothers how to assess non‐verbal pain cues, how to position the child safely to prevent aspiration, and how to manage breakthrough pain medications at home, which gives them a sense of meaningful contribution during their child's final days… (P12, Paediatric Nurse).

## 7 Category 3: Competencies in Psychological Care and Bereavement Support

This category encompassed the skills and attitudes needed for strengthening family resilience, providing care during bereavement, and the clinical judgment required for referring families to psychological support resources, highlighting that EOL care extends beyond the child's death.

### 7.1 Multi‐Stakeholder Perspectives and Contextual Nuances

The critical need to support families through anticipatory grief and post‐mortem bereavement was widely acknowledged across all participant groups. However, the approaches highlighted distinct dimensions reflecting their respective positions. Clinical nurses identified themselves as the providers of immediate emotional first‐aid, actively assessing when to triage and refer families for specialized psychological help. Nursing supervisors viewed this category systemically, focusing on establishing post‐loss follow‐up protocols and mitigating staff burnout. Faculty members emphasized the theoretical need to integrate advanced grief models into nursing curricula. Parents, conversely, highlighted the profound solace of unconditional presence—having nurses who provided a safe space to mourn and listened without judgment after their child's passing.

#### 7.1.1 Applying Psychological Support Skills to Enhance Family Resilience

This subcategory reflects nurses' skills in enhancing families' capacity to cope with the psychological distress associated with the child's illness and to prepare them for emotional challenges. Through the attitude of emotional support and the skill of teaching adaptive coping strategies, nurses helped families manage stress and emotional reactions. A paediatric nurse stated:

…Parents often carry tremendous guilt, feeling they somehow failed their child. Our role is to provide consistent psychological reassurance, validate their caregiving efforts, and guide them through deep breathing and grounding techniques when acute panic takes over… (P10, Paediatric Nurse).

#### 7.1.2 Demonstrating Supportive Interpersonal Skills During Family Bereavement

This subcategory refers to nurses' skills in providing psychological and emotional support of families during the bereavement period following the child's death. According to participants, nurses facilitated coping with loss through the attitudes of empathy, the skill of active listening and creating a safe space for families to express their emotions. A nursing supervisor reflected:

…Care does not terminate when the cardiac monitor stops. Bereavement care requires nurses to maintain a serene environment, assist parents in holding or bathing their child for the last time if they wish, and listen empathetically without offering clichéd platitudes… (P6, Nursing Supervisor).

A bereaved parent expressed the lasting impact of this care:

…The nurses treated my deceased child with such gentleness and respect, wrapping him tenderly and staying with us while we wept. That compassionate image gave us immense comfort during our darkest hours… (P17, Bereaved Mother).

#### 7.1.3 Assessing Needs and Navigating Professional Boundaries

Participants highlighted nurses' clinical and ethical judgment in assessing families' complex psychological needs and navigating professional boundaries. Recognizing the limits of their own interventions, competent nurses utilize their knowledge of available support systems to facilitate timely referrals to specialized mental health resources, such as counsellors, psychologists, or peer support groups. The purpose of these referrals was to ensure access to professional interventions and continued psychological support. An oncology nurse elaborated:

…When we observe signs of severe depressive withdrawal, severe trauma responses, or parental relationship breakdown, we recognize the boundary of nursing care and promptly refer the family to our clinical psychologist or social work team for specialized, ongoing intervention… (P7, Paediatric Oncology Nurse).

## 8 Category 4: Competencies in Cultural and Spiritual Care

In paediatric EOL care, providing holistic support necessitates profound competence in addressing the existential and cultural realities of families. Data analysis revealed that spiritual and cultural care emerged clearly as an independent and foundational competency category. Nurses demonstrated that supporting a family's belief system is not a secondary or complementary task, but an ethical obligation embedded in daily practice.

### 8.1 Multi‐Stakeholder Perspectives and Contextual Nuances

All groups agreed that spiritual care must align with the family's specific cultural beliefs. However, bedside nurses focused on pragmatic accommodations, such as allowing space for bedside prayers, while parents highlighted the comfort derived from non‐judgmental acceptance of their rituals. In contrast, nursing supervisors emphasized the need for formal referral pathways to hospital clerics, and faculty members focused on the theoretical distinction between generic spiritual comfort and formal religious practices.

#### 8.1.1 Demonstrating Spiritual Care Skills to Facilitate Meaning‐Making and Comfort

Participants emphasized that fostering spiritual comfort relies heavily on the nurse's authentic presence and support for existential meaning‐making. Nurses described these actions as humanistic care. This competency involves facilitating spiritual practices such as prayer, honouring periods of silence, accommodating religious rituals at the bedside, and providing a calming, empathetic presence that validates the family's existential distress. A PICU nurse shared:

…In our cultural context, spiritual rituals provide families with ultimate solace. We actively encourage parents to recite sacred texts, play quiet recitations, or perform traditional prayers beside their child, ensuring our clinical activities do not intrude upon these sacred moments… (P11, PICU Nurse).

#### 8.1.2 Tailoring Care Plans to Families' Religio‐Cultural Beliefs, Modesty Traditions and Taboos

This subcategory highlights the nurse's capacity to dynamically align paediatric end‐of‐life care with the family's unique worldview, cultural values and religious frameworks. Beyond theoretical awareness, this competency demands an uncritical, non‐judgemental acceptance of cultural rituals, strict adherence to modesty practices, and keen navigation of prognostic communication taboos. Nurses navigate the delicate balance between conveying clinical reality and honouring cultural perspectives, such as avoiding blunt prognostic disclosures that may be perceived as extinguishing spiritual hope. A paediatric oncology nurse illustrated this balance:

…Every family processes illness through their own cultural lens. Some request specific religious figures or traditional modesty practices during EOL care; our professional duty is to accommodate these preferences without judgement… (P7, Pediatric Oncology Nurse).

A nursing faculty member further emphasized the importance of contextual communication:

…In certain cultural settings, direct prognostic pronouncements of imminent death are perceived as extinguishing spiritual hope. Competent nurses use nuanced, culturally sensitive language that conveys clinical reality while respecting the family's faith in spiritual healing… (P4, Nursing Faculty).

#### 8.1.3 Applying Clinical Judgement for Targeted Spiritual and Cultural Referrals

This subcategory refers to nurses' clinical and ethical judgment to identify families' spiritual needs and refer them to appropriate resources such as hospital chaplains, religious counsellors, or spiritual support groups. This requires a comprehensive knowledge of available support networks. The findings showed that such referrals are most effective when implemented within an interdisciplinary framework and coordinated with other members of the care team. A nursing supervisor noted:

…When parents grapple with existential questions such as ‘Why is God letting my child suffer?’, nurses must recognize their clinical limits and coordinate with hospital religious counselors or pastoral care services… (P6, Nursing Supervisor).

## 9 Category 5: Ethical and Advanced Clinical Decision‐Making Competencies

This category reflects nurses' ethical judgment and clinical skills when confronting complex paediatric EOL decisions, which are often made under conditions of uncertainty, emotional distress, and legal or cultural constraints. This category included three subcategories.

### 9.1 Multi‐Stakeholder Perspectives and Contextual Nuances

There was broad agreement among participants on prioritizing the child's dignity and minimizing non‐beneficial interventions. Despite this convergence, practical perspectives varied. Clinical nurses highlighted their role as bedside advocates and the moral distress of performing futile resuscitations. Nursing supervisors were primarily concerned with mediating conflicts between parental preferences and institutional or legal mandates. Faculty members framed this competency around formal bioethical deliberation models, whereas parents emphasized a profound need for transparent, uncoerced guidance when navigating complex EOL decisions.

#### 9.1.1 Demonstrating Facilitation Skills in Shared Decision‐Making

This subcategory emphasizes nurses' communication skills and clinical knowledge in facilitating shared decision‐making between families and the healthcare team. Participants reported that although the legal authority for medical decisions—such as Do Not Resuscitate (DNR) orders or treatment limitation—rests strictly with physicians and legal guardians, nurses play a critical role in supporting the decision‐making process. Their competency lies in preparing families emotionally and cognitively, clarifying medical information, and bridging communication between the family and the medical team. A paediatric nurse explained:

…Physicians often explain DNR orders and treatment limitations in dense medical terminology that overwhelms parents. We step in afterward to translate those concepts into understandable language, answer their unspoken questions, and ensure they feel supported rather than abandoned in making these profound choices… (P2, Paediatric Nurse).

#### 9.1.2 Applying Ethical Judgement and Conflict‐Resolution Skills in EOL Dilemmas

This subcategory refers to nurses' conflict‐resolution skills and ethical judgement to navigate ethical conflicts such as futile treatment, ineffective resuscitation and tensions between ethical principles and legal obligations. Nurses endeavoured to maintain a professional attitude while managing the psychological burden associated with these dilemmas. A PICU nurse shared:

…Performing chest compressions and aggressive interventions on a child with terminal, irreversible multi‐organ failure causes immense moral distress. Competent nurses must possess the ethical reasoning to advocate for palliative transitions and participate in interdisciplinary ethical deliberations to prevent non‐beneficial suffering… (P11, PICU Nurse).

#### 9.1.3 Applying Dignity‐Conserving Care Practices

This subcategory underscores the professional attitude and ethical judgement required for prioritizing the child's human dignity during the final hours and days of life. Respecting privacy, minimizing unnecessary invasive interventions, and ensuring the child is cared for respectfully were identified as indicators of this competency. A PICU nurse stated:

…In the final hours of life, our focus shifts entirely to dignity. This means minimizing unnecessary invasive monitoring, providing gentle hygiene, managing terminal secretions quietly, and ensuring the child is surrounded by family love rather than cold technical machinery… (P11, PICU Nurse).

A bereaved parent corroborated:

…Even when my daughter was unresponsive, the nurse spoke softly to her before touching her, repositioned her gently, and kept her covered and peaceful. That profound respect for her dignity meant everything to us… (P17, Bereaved Mother).

## 10 Category 6: Competencies in Interdisciplinary and Team‐Based Collaboration

The findings demonstrated that effective paediatric end‐of‐life care is not possible without strong collaboration among members of the healthcare team. This category comprised two subcategories.

### 10.1 Multi‐Stakeholder Perspectives and Contextual Nuances

Across the interviews, participants consistently highlighted that high‐quality paediatric EOL care relies on interprofessional collaboration. Clinical nurses focused on the mechanics of shift handovers and informal on‐the‐job peer learning. Nursing supervisors emphasized smoothing communication across different medical teams. In the narrative accounts of the participating parents, interprofessional coherence was experienced primarily as the need for a unified healthcare team to prevent the emotional fatigue of repeatedly recounting their child's clinical history.

#### 10.1.1 Demonstrating Collaborative Teamwork and Communication

This subcategory highlights nurses' skills and clinical judgement in demonstrating collaborative teamwork and effective communication to coordinate care among physicians, psychologists, chaplains, and other healthcare professionals. By organizing care processes, nurses helped prevent fragmented decision‐making and ensured coherent care delivery. A paediatric nurse noted:

…Managing a terminal pediatric case requires coordinating multi‐specialty inputs. Nurses ensure that pain consults, psychological support, and dietary modifications are synchronized into one cohesive daily care plan, preventing fragmented or contradictory care… (P15, Pediatric Nurse).

This interdisciplinary coordination was equally echoed by family members, who viewed the nurse as a crucial stabilizing anchor across fragmented clinical encounters:

…The hardest part was having different doctors and specialists come in at different hours, asking the same questions or giving slightly contradictory impressions. We felt immense relief when the bedside nurse acted as our anchor—updating the charts, speaking with the attending physicians, and making sure everyone was on the exact same page so we didn't have to keep explaining our child's state over and over again… (P16, Bereaved Mother).

#### 10.1.2 Demonstrating Effective Information Exchange Skills for Continuity of Care

This subcategory highlights nurses' communication skills and professional attitudes in the timely and accurate sharing of clinical, psychological, and family‐preference information. This is particularly important during shift handovers and team communication to maintain the continuity of care. A clinical nurse emphasized:

…During shift handovers, it is not enough to report vital signs and medication dosages. We must clearly convey the family's emotional trajectory, their specific spiritual preferences, and agreed goals of care so the incoming nursing shift maintains unbroken, individualized continuity… (P14, Pediatric Nurse).

## 11 Discussion

To critically interpret the findings beyond a purely descriptive inventory of competencies, it is valuable to examine the dialectic tension between existing clinical realities and the normative aspirations articulated across stakeholder groups. While our primary inductive analysis generated six thematic competency categories grounded directly in the data, a cross‐stakeholder synthesis offers a tentative conceptual framework delineating four intersecting dimensions of paediatric EOL competencies:

First, competencies currently enacted in practice, primarily reflected in bedside nurses' narratives and corroborated by parental accounts (spanning Categories 1 and 3), largely encompass foundational physical comfort care, intuitive emotional empathy, and the respectful facilitation of cultural or religious rituals. As our findings suggest, these everyday bedside actions are frequently sustained by nurses' individual moral resilience and compassion rather than systematic institutional preparation.

Second, competencies perceived as clinical gaps, explicitly acknowledged by frontline clinicians during active patient deterioration (predominantly in Categories 2, 3 and 5), include advanced pharmacological symptom titration, navigating high‐stakes ethical dilemmas (such as prognostic disclosure and DNR conflicts), and delivering structured post‐loss bereavement care.

Third, normative and aspirational competencies, predominantly articulated by nursing faculty and nurse supervisors (reflecting Categories 4, 5 and 6), represent an ideal target model of what comprehensive paediatric palliative nursing ought to entail—emphasizing professional advocacy, interdisciplinary coordination, and ethical leadership.

Finally, resource‐ and education‐dependent competencies cut across these dimensions as advanced capabilities whose routine execution remains fundamentally contingent on structural reforms. Without formal integration of paediatric palliative care into the national nursing curriculum and the establishment of dedicated supportive infrastructure (such as specialized hospices and institutional bereavement services), these aspirational capabilities will remain theoretical expectations rather than consistently operationalized clinical practices.

Framing the findings through this tentative four‐fold conceptual synthesis clarifies why substantial discrepancies persist between what frontline nurses can practically deliver under acute systemic constraints and what educational and managerial standards demand.

Paediatric EOL care is recognized as one of the most challenging areas of healthcare due to its clinical, ethical, cultural and emotional complexities, particularly in countries such as Iran where paediatric palliative care has not yet been fully developed. In such contexts, nurses play a pivotal role in delivering humane, ethically oriented and family‐centred care. Accordingly, the present study aimed to explore the competencies perceived as important for nurses caring for children at the end of life. While previous frameworks often emphasized broad, multidimensional palliative care abilities—defining competency as a dynamic integration of knowledge, skills and caring attitudes (Worldwide Palliative Care Alliance and World Health Organization 2014)—our findings provide a more critical interpretation.

Importantly, in interpreting these findings, a clear distinction must be maintained between participants' reported experiences, normative stakeholder expectations and our conceptual synthesis. The clinical practices described as currently enacted reflect the concrete bedside experiences reported by bedside nurses and bereaved parents, rather than an objective audit of clinical practice across all settings. Conversely, the advanced competencies identified as lacking or aspirational predominantly capture the normative expectations articulated by academic educators and nurse managers, juxtaposed with frontline nurses' personal expressions of moral and clinical distress. Therefore, the primary contribution of this research does not lie in proving universal deficits across clinical practice, but rather in offering a context‐specific conceptualization of stakeholder‐perceived competencies within the sociocultural and acute clinical setting represented in this study, illustrating how care practices are shaped by participants' accounts of institutional constraints and familial dynamics.

Furthermore, while many of the identified competencies—such as empathetic communication and family support—may initially appear to align with general adult palliative care frameworks, this study offers crucial confirmatory value and further contextual insights specifically tailored to the paediatric context. Unlike adult palliative care, paediatric EOL situations involve profound emotional distress associated with the unnatural loss of a child, complex and surrogate parental decision‐making, and highly intricate family dynamics. Therefore, our findings translate general palliative principles into a highly specialized and emotionally charged environment. By conceptualizing these specific competencies, this study highlights how nurses must fundamentally adapt standard EOL care to meet the unique developmental, psychological, and familial needs present in paediatric settings, thereby bridging a critical gap in nursing literature.

When compared with established international frameworks—such as the European Association for Palliative Care (EAPC) paediatric core competencies and the Education in Palliative and End‐of‐Life Care for Paediatrics (EPEC‐Paediatrics) curriculum—our findings demonstrate both areas of convergence and context‐specific extensions. The essential categories identified herein, including holistic symptom alleviation, honest yet empathetic communication, and active family engagement, mirror core tenets globally recognized as foundational to paediatric end‐of‐life care (Downing et al. 2014; Widger et al. 2018).

However, this study provides potentially distinctive, context‐informed insights into how these competencies are operationalized within healthcare environments lacking formalized paediatric hospice programs or institutionalized bereavement support systems. In such settings, nurses do not merely deliver standardized palliative protocols; rather, they must navigate complex ethical dilemmas shaped by family communication patterns and spiritual coping practices described by participants. Furthermore, while international models often presuppose institutional support for staff, our participants demonstrated self‐generated emotional adaptation strategies amid moral distress. Thus, these findings capture not only widely recognized expectations but also the localized, socio‐emotional adaptations perceived as necessary in developing palliative care systems.

When comparing our findings with existing literature, several competencies align closely with established global standards, suggesting broad, cross‐contextual relevance. For instance, competencies in comprehensive physical care and empathetic communication are foundational regardless of the setting. Similar to international findings which emphasize the balanced use of pharmacological and non‐pharmacological interventions (Arora et al. 2021; Cheraghi et al. 2016; Farrag et al. 2023), nurses in our study prioritized symptom management to enhance the child's remaining quality of life. Furthermore, consistent with previous studies on communication (Barrett et al. 2023; Chong et al. 2019; Clark et al. 2014; Taheri‐Ezbarami et al. 2024), parents consistently require empathy and honest information, particularly when breaking bad news. In terms of psychological care, our findings align with competencies endorsed internationally, such as those by the Education in Palliative and End‐of‐Life Care‐Paediatrics (EPEC‐Paediatrics) curriculum, which emphasize strengthening family resilience and providing structured bereavement support (Postier et al. 2022). Empowering families through practical education about anticipated physical changes represents a widely recognized approach for alleviating parental distress (Das et al. 2021).

However, while these core palliative skills share broad international relevance, their operationalization in Iran reflects distinct socio‐cultural complexities. A key insight highlighted by this study is how cultural and spiritual care is deeply intertwined with clinical and ethical decision‐making rather than functioning as an adjunct service. While healthcare systems globally exhibit diverse approaches to integrating spirituality and autonomy, our findings illustrate how nurses in this specific socio‐cultural context actively navigate families' religious coping strategies, modesty norms, and faith‐based perspectives on illness and divine will. In the present sociocultural setting, participants described how specific faith‐based perspectives and spiritual coping strategies shaped some families' perceptions of illness, suffering and prognosis (Banazadeh et al. 2015; Barratt et al. 2024). In several accounts, families' expressions of hope for recovery or spiritual healing influenced their readiness to engage with palliative transitions (Jeong et al. 2024; Lee et al. 2024; Wiener et al. 2013). Accordingly, nurses in this study described navigating sensitive communication to convey clinical reality while respectfully acknowledging the family's expressed faith‐based perspectives (Eshghi et al. 2025; Shorey and Chua 2022), avoiding any assumption that all families approach end‐of‐life care in a uniform manner. Although previous local studies note implementation barriers in spiritual care, our findings underscore that mediating these ethical‐religious tensions serves as an essential adaptive professional capability for nurses practicing in this setting.

Another stark contrast between our findings and international literature relates to the clinical environment and infrastructure. Established international frameworks often assume the presence of specialized paediatric hospices, home‐based palliative services, and dedicated interdisciplinary teams. In contrast, our study revealed that most paediatric EOL care in Iran occurs within acute hospital settings, particularly PICUs and oncology wards. Because formal palliative infrastructure is largely absent, critical care and oncology nurses are forced to adopt specialized palliative roles. Consequently, while structured paediatric palliative care curricula have been increasingly integrated into nursing education across several high‐resource healthcare systems, literature continues to document a substantial gap in formal training programs (Piette et al. 2021; van Teunenbroek et al. 2024). Therefore, in the absence of structured training, nurses must rely heavily on adaptive communication skills and ethical resilience developed on the job within acute care settings (Iranmanesh et al. 2014; Rassouli and Salmani 2019).

This lack of infrastructure also exacerbates stakeholder disagreements, adding another layer of complexity to advanced clinical decision‐making. While international studies consistently highlight the importance of personal choice and parental participation in shared decision‐making (Beaussant et al. 2021; Pravin 2023), decision‐making in the study setting was described by participants as operating within specific legal and institutional frameworks, where formal medical authority (such as DNR orders or treatment limitation) is situated within the jurisdiction of physicians and legally recognized guardians. Rather than reflecting an immutable characteristic across all Islamic healthcare settings, these dynamics highlight the localized legal and institutional realities that bedside nurses must navigate when supporting families through difficult end‐of‐life decisions. Therefore, unlike models that emphasize autonomous nursing decisions, our findings highlight nurses in a crucial mediating role: they bridge the communication gap, clarify medical realities, and manage ethical conflicts between physicians recognizing medical futility and parents demanding aggressive treatments (Miller et al. 2023).

Finally, it is critical to interpret these competencies within appropriate conceptual and methodological boundaries. While the identified competencies aim to facilitate dignified EOL care, the concept of a ‘good death’ remains an interpretive, highly subjective construct rather than a standardized outcome across cultures (Meier et al. 2016). Because this study did not measure clinical or bereavement outcomes, these competencies should be understood as foundational capacities that support a dignified passing rather than guaranteed predictors of a good death. Combining non‐pharmacological comfort measures with spiritual practices offers significant benefits; however, caution is warranted to ensure patient safety, informed consent, and alignment with nurses' legal scope of practice within hospital policies. Addressing these infrastructural and educational gaps through structured training programs remains essential for empowering nurses to navigate the delicate intersection of clinical, cultural, and ethical care at the end of life.

## 12 Strengths and Limitations of This Work

The study utilized multiple approaches to enhance the trustworthiness and rigour of the findings, following Lincoln and Guba's criteria. However, several limitations must be recognized.

Methodologically, the research was conducted in a single‐hospital clinical setting, which inherently results in cultural specificity and limited transferability of the findings to different environments. The clinical context also led to a possible overrepresentation of PICU and oncology experiences, as well as an inevitable overlap between palliative‐care and EOL experiences in participants' narratives.

Regarding the sample, the study relied on small and uneven stakeholder groups (*n* = 17). Specifically, the inclusion of only two parents restricts the depth of family‐centred insights. Given parents' extreme psychological vulnerability during paediatric EOL care, this subgroup remained intentionally small; consequently, there is a lack of independent saturation for the parent and supervisor subgroups. A notable limitation is the absence of children's perspectives. Due to the critical and unstable condition of paediatric patients at the end of life, their inclusion was ethically unfeasible, meaning interpretations of children's needs are mediated through adult caregivers. Moreover, there is a possible selection bias resulting from the mental‐health exclusion criterion. While ethically necessary, it may have excluded those experiencing the highest levels of moral distress, predominantly capturing the views of those with more robust coping mechanisms.

In terms of data collection and analysis, relying on self‐reports introduces possible social‐desirability bias. Furthermore, prior familiarity between the researcher and some participants creates potential interviewer–participant power relationships that may have unintentionally impacted the provided answers. Additionally, because interviews were conducted in Persian, there is a risk of translation‐related loss of meaning during the coding and reporting processes.

Finally, because this qualitative design explores subjective perceptions, it inherently reflects an inability to assess actual nursing competence or care outcomes. The developed framework represents stakeholder expectations rather than objectively measured clinical proficiency.

## 13 Recommendations for Further Research

Future research is recommended to examine paediatric EOL nursing competencies using mixed‐methods or quantitative designs to validate and expand the findings of this qualitative study. Comparative studies across different regions, care settings (hospital, home‐based and community care), and cultural contexts may provide deeper insights into context‐specific and transferable competencies. In addition, interventional studies evaluating the effectiveness of structured educational programs and competency‐based training models in paediatric EOL care are warranted. Furthermore, future studies would benefit from a larger and more balanced representation of stakeholders, including active parental participation, to further explore potential nuances in expectations and experiences between parents and healthcare professionals.

Future research should prioritize exploring ethically sound and developmentally appropriate methods to capture the direct perspectives of older children and adolescents facing life‐limiting illnesses. Integrating their firsthand preferences regarding comfort, communication and spiritual support will be important for developing more inclusive and patient‐centred paediatric palliative care frameworks.

## 14 Implications for Policy and Practice

Consistent with the preliminary, context‐specific nature of this study, the stakeholder‐identified competencies should not be viewed as an established basis for direct clinical implementation. Rather, they provide foundational, exploratory insights that may prove potentially useful for informing the future, structured development of paediatric palliative care frameworks, curricula and institutional initiatives once subjected to further empirical validation:

- *Clinical practice and protocol development*: The identified competencies may serve as an initial conceptual reference to inform the future co‐design and refinement of localized clinical protocols. Rather than providing ready‐to‐implement standardized guidelines, these findings highlight essential practice dimensions—such as family‐centred communication and ethical deliberation during end‐of‐life care—that warrant inclusion in future guideline development initiatives following rigorous multi‐centre validation.
- *Curricular and educational design*: In high‐resource and developing settings alike, these context‐grounded findings offer valuable preliminary markers for educators seeking to conceptualize future paediatric palliative care training modules. Future continuous professional development programs could draw upon these perceived competencies to explore tailored competencies, specifically targeting moral distress navigation and complex parent‐provider communication, provided they are contextualized to local educational standards.
- *Institutional and policy considerations*: At an organizational level, these findings illuminate the profound ethical‐emotional tensions experienced by bedside nurses. While institutional policy decisions require broader system‐level evaluations, healthcare leaders can utilize these qualitative insights to recognize the necessity of exploring, piloting and evaluating formal workplace support mechanisms (e.g., structured reflective debriefing) tailored to paediatric and intensive care units.
- *Assessment tools and measurement frameworks*: Finally, while these exploratory qualitative competency areas provide an initial conceptual foundation, translating them into robust psychometric assessment tools requires a rigorous, staged validation process. Methodologically, qualitative item and domain generation should be followed by formal content and face validation via expert panels, preliminary pilot testing, construct validation (e.g., exploratory and confirmatory factor analyses), and comprehensive reliability testing before clinical or educational implementation, as demonstrated in recent paediatric nursing scale development literature (Cao et al. 2025). Such validated instruments will be essential for systematically evaluating paediatric nurses' educational needs and clinical competencies across diverse healthcare contexts.

## 15 Conclusion

To care for children and families at the end of life, nurses require a broad range of competencies that encompass not only clinical skills but also ethical considerations and context‐specific approaches. The multidimensional competencies identified through stakeholder perspectives in this research provide a preliminary, context‐specific conceptual account of perceived nursing capabilities. While these findings establish a groundwork that could eventually inform educational programs and clinical guidelines, they should be viewed as recommendations for further development and testing rather than tools for immediate implementation. Before these proposed competencies can be utilized as a formal framework, they must undergo rigorous stakeholder and expert content validation, as well as assessment across multiple hospitals and regions. Future research should prioritize the inclusion of broader parent and, where appropriate, child perspectives. Ultimately, translating this conceptual account into practice will require the behavioural definition of each competency and robust psychometric or performance‐based evaluation to ensure nurses are effectively empowered to care for terminally ill children.

## Author Contributions

**Latif Panahi:** conceptualization, investigation, funding acquisition, methodology, validation, visualization, software, formal analysis, project administration, data curation, supervision, resources, writing – review and editing, writing – original draft. **Zahra Taheri‐Ezbarami:** conceptualization, investigation, funding acquisition, writing – original draft, visualization, validation, methodology, writing – review and editing, project administration, resources, supervision, data curation, formal analysis. **Maryam Rassouli:** conceptualization, investigation, funding acquisition, writing – original draft, methodology, validation, visualization, writing – review and editing, formal analysis, project administration, data curation, supervision, resources. **Somaye Pouy:** data curation, supervision, resources, project administration, formal analysis, software, methodology, validation, visualization, writing – review and editing, writing – original draft, funding acquisition, investigation, conceptualization.

## Funding

This article is derived from an approved research project (Research number: 1403080711) funded by the Social Determinants of Health Research Center and Deputy of Research and Technology of Guilan University of Medical Sciences.

## Ethics Statement

This study was approved by the Ethics Committee of Guilan University of Medical Sciences (IRB approval: IR.GUMS.REC.1403.497, dated 22 January 2025). Prior to data collection, the study objectives were explained to all participants, and written informed consent was obtained. Participation was voluntary, and participants were informed of their right to withdraw from the study at any stage without any consequences. Confidentiality of information and anonymity of data were maintained throughout data collection, analysis and reporting.

## Conflicts of Interest

The authors declare no conflicts of interest.

## Supporting information


**Appendix S1:** COREQ (COnsolidated criteria for REporting Qualitative research) checklist.

**Appendix S2:** Semi‐structured interview guide.

## Data Availability

The data that support the findings of this study are not openly available due to reasons of sensitivity, human privacy and ethical restrictions (e.g., interview transcripts contain highly sensitive and emotional narratives regarding paediatric end‐of‐life care and the family grieving process). Anonymized excerpts of the data are available from the corresponding author upon reasonable request and subject to ethical approval.
