# Supplementary material for: Exploring Stakeholders' Perspectives on Required Nursing Competencies in Paediatric End‐of‐Life Care: A Qualitative Study
Source: Nurs Open. 2026 Nov 3;13(11):e71011. doi: 10.1002/nop2.71011 (PMC13730262; doi:10.1002/nop2.71011)
Supplement: Supplementary file 2 [file NOP2-13-e71011-s002.docx]

**Appendix S2. Semi-Structured Interview Guide**

**A. Interview Questions for Nursing Supervisors**

1. Drawing from your expertise, what are the key responsibilities of a skilled nurse in providing end-of-life care for a pediatric patient?
2. Would you please describe your experience providing care for a child nearing the end of life?
3. During that experience, which nursing interventions proved most beneficial for the child and their family?

**B. Interview Questions for Nurses**

1. Could you please elaborate on the pediatric end-of-life care services that nurses offer?
2. What competencies are essential for nurses delivering pediatric end-of-life care?
3. What recommendations do you propose for enhancing the proficiency and capabilities of nurses in delivering pediatric end-of-life care?
4. What elements contribute to the necessary nursing competencies for pediatric end-of-life care?

**C. Interview Questions for Nursing Faculty Members**

1. What forms of nursing care have you witnessed for pediatric patients nearing the end of life?
2. Could you please provide a more detailed explanation of your evaluation regarding the current standard of end-of-life care provided by nursing professionals?
3. What skills are essential for nurses in providing end-of-life care?
4. What recommendations do you have for enhancing nurses' skills and competencies in delivering end-of-life care?
5. What are the primary factors influencing the crucial competencies required for pediatric end-of-life nursing?

**D. Interview Questions for Bereaved Parents**

1. Could you please elaborate on the interventions nurses employed during your child's care?
2. Which of these elements of care provided the most benefit or utility for you and your child?

**E. Probing Questions**

To enhance the data and offer a more comprehensive understanding of the participants' experiences, the following probing questions were posed when appropriate.

1. Would you be able to provide additional details?
2. Could you provide an instance of this experience?
3. Could you describe your emotional response to this experience?
4. Would you be so kind as to provide further details regarding...?
5. Could you please clarify what you meant by your previous statement?
6. Could you elaborate further on this subject?
7. Could you please elaborate on any pertinent experiences or specific incidents you recall?
